# Effect of flow angle and flow profile on phase contrast flow measurements: overestimation at extreme angles and skewed profiles

**DOI:** 10.1186/1532-429X-11-S1-O53

**Published:** 2009-01-28

**Authors:** Kevin K Whitehead, Ravi Doddasomayajula, Matthew A Harris, Matthew J Gillespie, Mark A Fogel

**Affiliations:** Children's Hospital of Philadelphia, Philadlephia, PA USA

**Keywords:** Congenital Heart Disease, Imaging Plane, Flow Measurement, Pulsatile Flow, Straight Tube

## Introduction

Flow measurements derived from phase contrast velocity mapping (PC-MRI) have become an important part of pediatric cardiology, allowing accurate quantification of shunts, valve regurgitation, and cardiac output. Clinicians generally try to align the imaging plane orthogonal to flow. However, while flow velocities decrease by the cosine of the angle from orthogonal, the area should ideally increase by the same rate. There are situations in which it may be desirable to quantify flow in a vessel on an image that was not intended during the acquisition. In addition, in some flow regimes with highly skewed flow it is sometimes difficult to align the imaging plane with flow. Understanding the settings in which PC-MRI is accurate is important to the evaluation of congenital heart disease.

## Purpose

The purpose of this in vitro investigation is to assess the accuracy of PC-MRI flow measurements as a function of the angle of the imaging plane to the direction of flow. We hypothesize that the imaging angle does not have a significant effect on flow measurements.

## Methods

Both steady and pulsatile flows (0.7–10 L/min) were driven through an in vitro flow phantom consisting of tube diameters ranging from 10 mm to 19 mm both in a straight tube configuration and just distal to a 90 degree bend to simulate skewed flow. Through-plane PC-MRI velocity maps were obtained at each flow rate (1.5 T Siemens Avanto) with the imaging plane oriented from 0 to 75 degrees (15 degree increments). Actual flows were measured for each condition using an MRI-compatible calibrated ultrasound flowmeter (Transonics) interfaced to a PC. Flow was measured using off-line analysis (Argus, Siemens). Normalized flows (measured flow/actual flow) were compared to the angle from orthogonal to flow for all flow conditions. Variances in flow measurements between angles were compared using an F-test.

## Results

**Figure 1:**
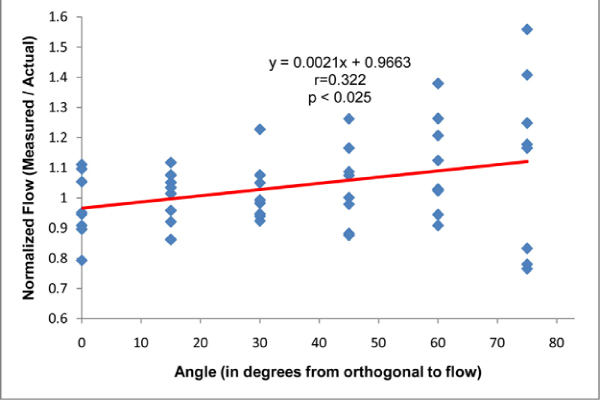
**Normalized flow vs imaging angle**.

Normalized flow demonstrated weak but significant correlation with flow angle for both steady and pulsatile flow conditions (p < 0.05). For pulsatile flow, mean normalized values ranged from 0.97 for 0 degrees to 1.12 for 75 degrees, and overestimation was as high as 11% for 0 degrees to as high as 56% for 75 degrees. Variability also tended to increase as angle increased, and the variance for 75 degrees was significantly greater than 0, 15, and 30 degrees. The flow estimation was not significantly different for skewed flow (distal to elbow) compared to flow in straight tubes. Figure 1.

## Conclusion

While PC-MRI is fairly robust with respect to flow angle, overestimation of flow increases significantly as a function of flow angle, to as high as a mean of 12% for the 75 degree angle. This is counterintuitive, and suggests that in general the measured velocity is not decreasing to the same degree that area is increasing. In addition, the variability of the measurements increases with increasing flow angle, especially at larger flow angles. The etiology of the error is likely related to partial volume effects. Care should be taken to be as orthogonal to flow as possible during PC-MRI to avoid overestimating flow.

